# Neuronal P2X4 receptor may contribute to peripheral inflammatory pain in rat spinal dorsal horn

**DOI:** 10.3389/fnmol.2023.1115685

**Published:** 2023-03-09

**Authors:** László Ducza, Andrea Gajtkó, Krisztina Hegedűs, Erzsébet Bakk, Gréta Kis, Botond Gaál, Roland Takács, Péter Szücs, Klára Matesz, Krisztina Holló

**Affiliations:** Department of Anatomy, Histology and Embryology, Faculty of Medicine, University of Debrecen, Debrecen, Hungary

**Keywords:** inflammatory pain, spinal dorsal horn, P2X4 receptor, central sensitization, primary afferents, glial cells, dorsal root ganglia

## Abstract

**Objective:**

Intense inflammation may result in pain, which manifests as spinal central sensitization. There is growing evidence that purinergic signaling plays a pivotal role in the orchestration of pain processing. Over the last decade the ionotropic P2X purino receptor 4 (P2X4) got into spotlight in neuropathic disorders, however its precise spinal expression was scantily characterized during inflammatory pain. Thus, we intended to analyze the receptor distribution within spinal dorsal horn and lumbar dorsal root ganglia (DRG) of rats suffering in inflammatory pain induced by complete Freund adjuvant (CFA).

**Methods:**

CFA-induced peripheral inflammation was validated by mechanical and thermal behavioral tests. In order to ensure about the putative alteration of spinal P2X4 receptor gene expression qPCR reactions were designed, followed by immunoperoxidase and Western blot experiments to assess changes at a protein level. Colocalization of P2X4 with neuronal and glial markers was investigated by double immunofluorescent labelings, which were subsequently analyzed with IMARIS software. Transmission electronmicroscopy was applied to study the ultrastructural localization of the receptor. Concurrently, in lumbar DRG cells similar methodology has been carried out to complete our observations.

**Results:**

The figures of mechanical and thermal behavioral tests proved the establishment of CFA-induced inflammatory pain. We observed significant enhancement of P2X4 transcript level within the spinal dorsal horn 3 days upon CFA administration. Elevation of P2X4 immunoreactivity within Rexed lamina I-II of the spinal gray matter was synchronous with mRNA expression, and confirmed by protein blotting. According to IMARIS analysis the robust protein increase was mainly detected on primary afferent axonterminals and GFAP-labelled astrocyte membrane compartments, but not on postsynaptic dendrites was also validated ultrastructurally within the spinal dorsal horn. Furthermore, lumbar DRG analysis demonstrated that peptidergic and non-peptidergic nociceptive subsets of ganglia cells were also abundantly positive for P2X4 receptor in CFA model.

**Conclusion:**

Here we provide novel evidence about involvement of neuronal and glial P2X4 receptor in the establishment of inflammatory pain.

## 1. Introduction

Pain is a striking and devastating symptom of many diseases affecting the physical and mental well-being (Noehren et al., 2015; Raffaeli et al., 2021). Several lines of evidence support that the main causating agents are inflammatory factors, malignant disorders as well as damages to the nervous system (Sica et al., 2019; Troiani et al., 2019). The condition may emerge in the form of *allodynia, hyperalgesia* or spontaneous pain states, in which the nociceptive threshold is markedly altered resulting in central sensitization (Majedi et al., 2018; Davies et al., 2019; Kuner and Kuner, 2021). Earlier findings retrieved from animal models confirmed the activation of peripheral afferents reacting to chemical stimuli released by immune cells, keratinocytes and endothel cells (Ji et al., 2016; Eitner et al., 2017). Till date much effort has been made to gain a better understanding of the pathological mechanisms including peripheral plasticity, transduction, and propagation of noxious inputs (Julius, 2013; Arcourt and Lechner, 2015; Bennett et al., 2019). In addition, the role of glial cells received growing attention lately in the central sensitisation (Salter and Beggs, 2014; Chen et al., 2018). Owing to the complicated molecular machinery involved in pain conditions, the treatment strategy is still a challenging health issue (Murray and Lopez, 2013). Purinergic P2X ATP sensitive ligand-gated ionotropic receptors are trimeric non-selective cation channels assembled from seven different homomeric and heteromeric subunits (P2X1–P2X7) encoded by different mammalian genes. P2X4 receptor is expressed by several cell types, involved in various physiological processes such as epithelial transport, metabolism, liver regeneration. The receptor is permeable to Na^+^, K^+^, and Ca^2+^ ions, and its activation upon ATP-binding induces cell depolarisation (Suurväli et al., 2017). In accordance with literature P2X4 receptor highly contributes to neuropathic pain. Overexpression of P2X4 is associated with nonadaptive modification of synaptic strength leading to central sensitisation (Suurväli et al., 2017; Long et al., 2018; Zhang et al., 2020). Earlier studies reported that in neuropathic pain P2X4 was mostly expressed by spinal microglial cells. Upon activation microglial cells initiate many signaling pathways *via* kinase cascades, which result in the secretion of variety of molecules that ultimately aggravate pain (Tsuda, 2016; Teng et al., 2019). Moreover, P2X4 may alter activity *via* neuron-microglial interactions (Zhang et al., 2020). Despite its unequivocal role in microglial cells, abundant neuronal P2X4 expression was also detected earlier at a spinal level (Lê et al., 1998), however evidence is still lacking on its distribution in DRG neurons and superficial spinal dorsal horn, even if previous works has highlighted the potential of neuronally expressed P2X4 receptor (Ulmann et al., 2010; Lalisse et al., 2018; Duveau et al., 2020). Therefore, the present study aimed to (i) describe the P2X4 expression in lumbar DRG and superficial spinal dorsal horn at a gene-and protein level, (ii) screen the potential receptor expressing sites here upon peripheral inflammation.

## 2. Materials and methods

### 2.1. Animals

The concept has been approved by the Animal Welfare Committee of the University of Debrecen (licence number: 212/2015/DEMÁB) in agreement with the national laws and European Union regulations (Directive of 24 November 1986 (86/609/EEC, European Communities Council). Animals were kept under standard *ad libitum* feeding conditions. Experiments were conducted on adult (3–4 months, weighing 250–300 g) male Wistar-Kyoto rats (Gödöllő, Hungary), among which two groups, the control (*n* = 21) and CFA treated (*n* = 18) animals were utilized. Increased focus was placed on minimizing animal use in our experimental design, therefore care was taken to follow the Three Rs (3Rs) guiding principles of animal experimentation. In CFA treated rats peripheral inflammatory pain was induced by administering injection of 100 μL 1:1 mixture of physiological saline and CFA agent (Sigma-Aldrich, St Louis, USA, catalog no.: F5881) into the right hindpaw described earlier by Hylden et al. (1989). Based on the latest terminology of International Association of the Study of Pain (IASP^1^) our experimental design is interpreted as one of the nociceptive pain models.

### 2.2. Mechanical allodynia test

Measurement of mechanical allodynia were carried out on 3 control and 3 CFA-treated animals. Rats were exposed to noxious mechanical stimulus to evaluate hindpaw withdrawal reflex. Mechanical threshold was tested by modified von Frey test (Dynamic Plantar Aesthesiometer, Ugo Basile, Gemonio, Italy). Following a 20-min habituation in a cage covered with acrylic sidewalls and mesh floor, a flexible, von Frey-type filament was directed with gradually increasing force on the plantar surface of the animal hindpaw until withdrawal. Mechanical withdrawal threshold (MWT) of both hindpaws was recorded prior to CFA injection, then repeated daily upon CFA administration. The measurement was replicated five times for each paw alternating between the left and right hindpaw.

### 2.3. Thermal allodynia test

Experiments were carried out on 3 control and 3 CFA-treated animals. Rats were placed into a plastic cage with a Perspex enclosure that rendered the animals unrestrained for the duration of the measurement. Temperature threshold was tested by Plantar Test Instrument-Hargreaves Apparatus (Ugo Basile, Genomio, Italy). Following a 20-min habituation the hindpaw of the rats were positioned above an infrared light source, directed onto the plantar surface until the animal withdrew its paw. Thermal withdrawal latency (TWL) of both hindpaws was recorded prior to CFA injection, then repeated daily upon CFA administration. The measurements were repeated five times in each case alternating between the left and right hindpaw.

### 2.4. Quantitative real-time PCR analysis (RT-qPCR)

#### 2.4.1. RNA isolation and reverse transcription

Experiments were carried out on 3 control and 3 CFA-treated rats. Animals were euthanized 3 days after CFA administration with intraperitoneally injected sodium pentobarbital (50 mg/kg). Control animals were handled similarly, though without CFA injection. L4-L5 spinal segments and lumbar DRG were removed, then immersed in RNAlater Stabilizing Solution (Thermo Fisher Scientific, Waltham, MA, USA, catalog no.: 00695052). Thereafter samples underwent flash freezing in liquid nitrogen, then storage at −80°C. Samples were mixed with TRIzol Reagent (Applied Biosystems, Foster City, CA, USA), then centrifuged at 10,000 g at 4°C for 15 min with a supplement of 20% RNase-free chloroform. Upon incubation in 1,000 μL RNase-free isopropanol for 1 h at −20°C, total RNA was purified in 70% ethanol. Finally, the RNA precipitate was resuspended in RNase-free water, then stored at −80°C. RNA purity and concentration were measured using a NanoDrop 1,000 spectrophotometer (Thermo Fisher Scientific). Reverse transcription was performed from 1,000 ng of total RNA using the High Capacity cDNA Reverse Transcription Kit (Thermo Fisher Scientific) according to the instructions provided by the manufacturer. The resulting cDNA was stored at −20°C.

#### 2.4.2. Quantitative PCR

RT-qPCR reactions were carried out by using the comparative ∆∆Ct method. As a relative quantification SYBR Green-based system (Promega, Madison, WI, USA) was applied. P2X4 primer pairs were designed by the Primer-BLAST service of the NCBI (National Institutes of Health) and ordered from Integrated DNA Technologies (IDT, Coralville, IA, USA). Nucleotide sequences of the used primer pairs are provided in *Table S1* in the Supporting information section. GoTaq qPCR Master Mix (Promega) was applied for the reactions that were performed with a QuantStudio 3 Real-Time PCR System (Thermo Fisher Scientific). As a standard thermal profile, initial denaturation (at 95°C, for 2 min), then 40 cycles of denaturation (at 95°C, for 5 s), annealing and extension (at 60°C, for 30 s), and final extension (at 72°C, for 20 s) were used. Following the 40 cycles of amplification, a melt curve stage was carried out involving 3 steps: denaturation at 95°C for 15 s, annealing at 55°C for 15 s, and a dissociation phase with 0.15°C/s increments between 55°C and 95°C. Acquired data were first analyzed using the QuantStudio Design and Analysis Software (version 1.5.1), then processed with Microsoft Excel programme. The ∆∆Ct method (Livak and Schmittgen, 2001) was used for data analysis, Ct values were normalized onto the most stably expressed reference gene out of four candidates and then treated and respective control values were compared. NormFinder software was used to determine the optimal normalization gene from our selection of four reference genes based on expression stability. In our case, RT-qPCR data were normalized to the expression level of RPL4 (*ribosomal protein L4*, Supplementary Table S1).

### 2.5. Immunohistochemistry

#### 2.5.1. Tissue preparation

Immunohistochemical experiments were carried out on 9 control and 9 CFA-treated rats. Animals were sacrificed as described in chapter 2.4 quantitative real-time PCR analysis. Subsequently, transcardial perfusion was conducted with oxygenated physiological saline solution (mixture of 95% O_2,_ 5% CO_2_) and fixative containing either 4% paraformaldehyde (single-and double imunolabelings) or 2.5% paraformaldehyde and 0.5% glutaraldehyde (electron microscopy). Thereafter, L4-L5 segments of spinal cord and lumbar DRG were collected and postfixed, then cryoprotected in 0.1 M PB solution containing 20% sucrose concentration overnight. Proper penetration of chemicals was granted by immersing spinal cord into liquid nitrogen. Agar-embedded samples were sectioned at 50 μm thickness with vibratome (Leica VT1000S, Leica Biosystems, Deer Park, IL 60010 United States).

#### 2.5.2. Single immunolabeling

Immunoperoxidase reactions were carried out on rat spinal dorsal horn sections of 3 control and 3 CFA injected animals. Prior to antibody treatments the sections were gently shaken in 0.01 M Tris-phosphate-buffered saline (TPBS, pH 7.4) solution supplemented with 10% normal goat serum (NGS) (Vector Labs, Burlingame, CA, USA, catalog no.: S-1000) for 50 min. Free-floating sections were incubated with anti-P2X4 antibody (1:1,000; Alomone Labs, Jerusalem BioPark, Israel catalog no.: APR-002) for 72 h at 4°C. Anti-P2X4-antibody was also diluted in TPBS to which 1% NGS was added. Thereafter, the sections were transferred into biotinylated goat anti-rabbit IgG solution (1:200; Vector Labs) for 4 h at room temperature. Afterwards, avidin-biotinylated horseradish peroxidase complex (1:100, Vector Labs) was transferred on the sections for 24 h at 4°C, then chromogen reaction was visualized with 3,3′-diaminobenzidine (DAB) reagent (Sigma-Aldrich, catalog no.: D-5637). Sections were mounted on glass slides and coverslipped with DPX medium (Sigma-Aldrich). Fluorescent micrographs were captured by Olympus CX-31 epifluorescent microscope equipped with Olympus DP-74 camera (both manufactured by Olympus Co. Ltd., Tokyo, Japan). Optical density of images was analyzed by ImageJ software (version 1.8.0, NIH). As a control, rabbit anti-P2X4 antibody (diluted as 1:1,000, Alomone Labs) specificity have been preliminarily verified in knockout animals by the manufacturer, but we similarly tested it on our samples by adding anti-P2X4 antibody to synthetic P2X4 peptide (Alomone Labs catalog no.: BLP-PR002) for antibody depletion. Briefly, synthetic blocking peptide was blended with antibody (equimolar 1 μg peptide/1 μg antibody ratio), stored at 4°C overnight, then centrifuged. Following incubation with the mixture for 72 h at 4°C, spinal cord sections were further transferred into biotinylated rabbit anti-goat secondary antibody dissolved in TPBS (diluted as 1:200, Vector Labs, USA) for 4 h at room temperature. Afterwards, the sections were treated similarly to the single immunostaining method. The preadsorption of blocking peptide to anti-P2X4 antibody abolished the specific immunolabeling (Supplementary Figure S1A). In addition, negative control reaction omitting the primary antibody against P2X4 receptor was also carried out (Supplementary Figure S1B). Negative control reaction omitting the primary antibody was also performed in lumbar DRG (Supplementary Figure S1C). Of note, original DAB chromogen labeled images were converted into monochromatic gray-scale images to enhance contrast.

#### 2.5.3. Double immunolabeling

Double immunolabeling protocols were performed on 3 control and 3 CFA treated animals to study the distribution of P2X4 on variety of neuronal and glial labelings including markers of primary afferents (IB4, CGRP), axonterminals of glutamatergic and γ-aminobutyric acid (GABA) ergic interneurons (VGLUT2, VGAT), postsynaptic densities of excitatory and inhibitory synapses (PSD95, Gephyrin), as well as astrocytes (GFAP) and microglial cells (Iba1) within spinal dorsal horn. Prior to antibody treatments the sections were gently shaken in phosphate-buffered saline (PBS, pH 7.4) solution supplemented with 10% NGS serum. Antibodies were also diluted in PBS to which 1% NGS was added. First, free-floating sections were incubated with an antibody mixture that contained rabbit anti-P2X4 antibody (1:1,000; Alomone Labs) and one of the antibodies as follows: (1) guinea pig anti-calcitonin gene-related peptide (CGRP, 1:2,000, Peninsula Labs, San Carlos, California, USA, catalog no.: T5027), (2) biotinylated isolectin B4 (IB4, 1:2000, Invitrogen, Eugene, Oregon, USA, catalog no.: I21414), (3) guinea pig anti-vesicular glutamate transporter 2 (VGLUT2, 1:2,000, Millipore, Temecula California, USA, catalog no.: AB2251), (4) mouse anti-vesicular gamma-amino butyric acid transporter (VGAT, 1:200, Synaptic Systems, Goettingen, Germany, catalog no.: 131011), (5) mouse anti-postsynaptic density protein 95 (PSD95, 1:100, Frontier, Geumcheon-Gu, Seoul, Korea, catalog no.: AB2723), (6) mouse anti-gephyrin (1:100, Synaptic Systems, catalog no.:147021) (7) mouse anti-glial fibrillary acidic protein (GFAP, 1:1,000, Millipore, Temecula, California, USA, catalog no.: MAB3402,), and (8) guinea-pig anti-ionized calcium binding adaptor molecule 1 (Iba1, 1:2,000; Synaptic Systems, Goettingen, Germany catalog no.: 234–004). Following incubation with primary antibody solutions for 72 h at 4°C, sections were transferred into proper mixture of secondary antibodies listed below: (a) goat anti-rabbit IgG-AlexaFluor 488 (1:1,000; Thermo Fisher Scientific, catalog no.: A11034) (b) goat anti-guinea-pig IgG-AlexaFluor 555 (1:1,000; Thermo Fisher Scientific, catalog no.:A21435) (c) streptavidin conjugated with AlexaFluor 555 (1:1,000, Invitrogen, Eugene, Oregon, USA, catalog no.: S21381) or (d) goat anti-mouse IgG-AlexaFluor 555 (1,1,000; Thermo Fisher Scientific, catalog no.:A21422). List of the applied primary and secondary antibodies are provided in *Table S2* in the Supporting information section. The protocol was slightly modified in stainings including PSD95 and gephyrin markers, before primary antibody incubation pepsin pretreatment was carried out for antigen retrieval. The sections were incubated for 10 min at 37°C in 0.2 M HCl containing 1 mg/ml pepsin (Watanabe et al., 1998). In control and CFA treated lumbar DRG tissue samples (L4-L5) double immunolabeling was performed by using antibodies raised against P2X4 receptor as well as CGRP peptidergic-and IB4-nonpeptidergic primary afferent markers.

#### 2.5.4. Quantification of confocal microscopy

The colocalization of applied markers with P2X4 was quantitatively analyzed on 1-μm thick single optical z-stack sections of the superficial spinal dorsal horn (Rexed laminae I–II) captured with Olympus FV3000 confocal laser microscope (60x oil-immersion lens, numerical aperture: 1.4). The confocal parameters (laser power, confocal aperture) were identical while recording each reaction. Scanned images were processed with Olympus Fluoview 2.1 and Adobe Photoshop CS6 softwares. Data were taken from three randomly selected sections of each animal. Identification of Rexed laminae I and II was based on the following: (a) The border between the dorsal column and the dorsal horn was detected based on the intensity of immunostaining. (b) The border between laminae II and III was estimated according to earlier descriptions (McClung and Castro, 1978; Molander et al., 1984). Briefly, double-fluorescent z-stack confocal sections were converted into rendered images by IMARIS (Bitplane ver.7.3) algorithm, with which immunoreactive spot/profile detection and colocalization were calculated. Volumes (μm^3^) of neuronal and glial profiles were determined from control and CFA treated samples, then P2X4 expression was quantified as the % volume of the marker-labeled structures containing P2X4-positive puncta / total volume of the markers. Our prior sample analysis showed that CFA-induced inflammation did not significantly alter the volumes of the investigated markers based on comparisons between control vs. CFA treated samples. Total number of CGRP and/or IB4 DRG cells was also quantified in sections taken from control and CFA treated samples, then checked whether they colocalize (in % number of the cells) with P2X4 receptor.

#### 2.5.5. Preembedding immunoperoxidase reaction for transmission electronmicroscopy

Preembedding immunostaining protocol was performed on sections taken from 3 control animals as described in chapter 2.5.2- single immunolabeling to visualize the ultracellular distribution of P2X4. After washing in 0.1 M PB (pH 7.4) and 1% sodium borohydride treatment for 30 min, free-floating sections were fixed with 2% paraformaldehyde and 0.5% glutaraldehyde. Prior to antibody treatments the sections were immersed in 10% NGS (Vector Labs) for 50 min. Anti-P2X4 antibody was diluted in 0.01 M TPBS to which 1% NGS was added. Upon incubation with rabbit anti-P2X4 antibody (1:1,000, Alomone Labs) for 72 h at 4°C, sections were placed into biotinylated goat anti-rabbit IgG (Vector Labs) for 4 h at 4°C. Following a treatment with avidin biotinylated horseradish peroxidase complex (Vector Labs) for 24 h at 4°C, the immunoreaction was visualized with 3,3′-diaminobenzidine (Sigma-Aldrich). Subsequently the sections were treated with 0.2% osmium-tetroxide for 50 min, dehydrated and then embedded into Durcupan ACM resin (Sigma-Aldrich, catalog no.: 44610) on glass slides. After reembedding, ultrathin sections were made and transferred on Formvar-coated single-slot nickel grids, and counterstained with uranyl acetate and lead citrate.

#### 2.5.6. Preembedding nanogold immunolabeling for transmission electronmicroscopy

Preembedding P2X4 nanogold immunostaining was performed on 3 control animals. Free-floating sections were handled identically as described in chapter 2.5.5 Preembedding immunoperoxidase reaction before secondary antibody treatment. Thereafter sections were transferred into a goat anti-rabbit IgG solution coupled with 1,4 nm gold particles (1:100, Aurion, Wageningen, The Netherlands) for 12 h at 4°C. Following washing steps in 0.01 M TPBS the sections were postfixed for 10 min in 2.5% glutaraldehyde, then washed repeatedly in 0.01 M TPBS and 0.1 M PB. Nanogold labeling was further intensified with silver enhancement (Aurion R-GENT, Wageningen, The Netherlands). Sections were treated with 1% osmium-tetroxide for 45 min, then dehydrated and embedded into Durcupan ACM resin (Sigma-Aldrich) on glass slides. Selected sections were reembedded, ultrathin sections were cut and placed on Formvar-coated single-slot nickel grids, and counterstained with uranyl acetate and lead citrate.

### 2.6. Western blotting

L4-L5 spinal segments of 3 control and 3 CFA injected animals were harvested. Detergent compatible BCA assay (Pierce, Rockford, USA) was used to measure protein concentration. Samples were dissolved in reducing buffer (50 μg protein/lane) and run on 12% SDS-polyacrylamide gels previously described by Laemmli (1970). Following separation proteins were electrophoretically transferred onto PVDF membrane (Millipore, Bedford, USA). The membranes were blocked with 10% normal bovine serum albumin (BSA, Sigma-Aldrich) in^2^ Tween-Tris-buffered saline (TTBS solution, 20 mM TRIS, 500 mM NaCl, pH 7.5, 0.05% Tween-20). Membranes were incubated with rabbit anti-P2X4 (1:1,000, Alomone Labs) and internal control antibody (mouse anti-β-tubulin, 1:2,000, Sigma-Aldrich) for 2 hours room temperature. Upon washing with TTBS, membranes were treated with goat anti-rabbit secondary antibody conjugated with horse-radish-peroxidase, (HRP, 1:200, DakoCytomation, Glostrup, Denmark) and goat anti-mouse secondary antibody conjugated with HRP (1:200, DakoCytomation). The labelled protein bands were visualized with DAB chromogen reaction (Sigma-Aldrich).

### 2.7. Statistics

Prior to the statistical analysis, sample sizes were not predetermined, but power tests were carried out to confirm that they were sufficient for quantifying the experimental data. Data were analyzed by SigmaStat software. Equal variances between data were presumed. Differences were considered significant when *p* < 0.05.Data sets of behavioral tests (*n* = 10/day) carried out in control and CFA treated animals were compared with compared with Repeated measures 2-way ANOVA followed by *Sidak’s* multiple comparison test. Data sets of qPCR analysis were determined by using nine parallel replicates (*n* = 9) taken from control and CFA treated animals, respectively. Inter-group statistical differences were determined by Student’s t-test. Mean-and standard deviation (SD) values were also calculated. Data sets of single immunolabeling were determined by using nine randomly selected sections taken from control and CFA treated animals respectively (3–3 animals, 3 sections 2 areas, *n* = 18). Between-groups statistical differences were calculated by Student’s t-test. Mean-and standard deviation (SD) values were also calculated. Data sets of double immunofluorescent labelings were determined by using nine randomly selected confocal sections (spinal dorsal horn and DRG samples) taken from control and CFA treated animals, respectively (3–3 animals, 3 sections 2 areas, *n* = 18). Statistical differences between control and CFA treated groups were determined by Student’s t-test. Mean-and standard deviation (SD) values were also calculated. Analysis of western blots and determination of the relative amount of P2X4 receptor protein were carried out on parallel replicates (n = 3) taken from control and CFA treated rats, respectively. Statistical differences between control and CFA treated groups were calculated by using Student’s *t*-test. Mean-and standard deviation (SD) values were also calculated.

## 3. Results

### 3.1. Animals showed significant mechanical nociceptive sensitivity during peripheral inflammation

**Figure 1:**
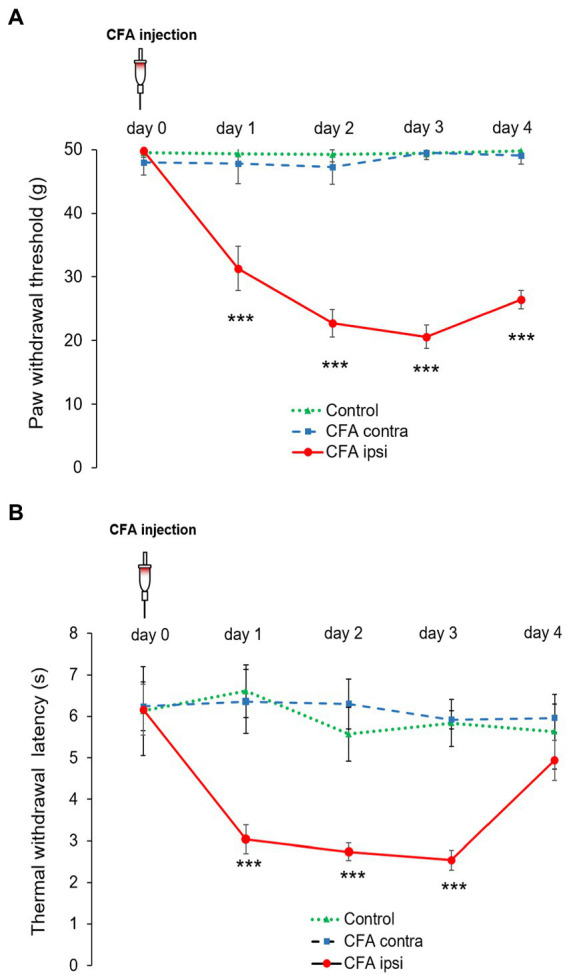
**(A)** Modified von Frey allodynia test illustrating mean mechanical paw withdrawal threshold (MWT) values on both hind limbs of control rats (Control, green dotted line with triangle) and rats administered with CFA (day 0) into the right (ipsilateral, CFA ipsi, red line with circle) hindpaw. The left hindpaw (contralateral, CFA contra, blue dotted line with square) was unhandled. Note that MWT values of control animals and the contralateral hind paw of CFA treated animals were parallel throughout the experimental design. CFA injection resulted in the highest decline in MWT on post-injection day 3, when MWT values dropped to 20.6 ± 1.85 g (***p < 0.001). The difference between data of the MWT obtained from control and CFA treated rats was also highly remarkable (***p < 0.001) during experimental day 1–3. Between-groups statistical differences were evaluated by using Repeated measures 2-way ANOVA followed by *Sidak’s* multiple comparison test Data are shown as mean + SD. **(B)** Thermal allodynia test indicating mean thermal withdrawal threshold latency (TWL) figures on both hind limbs of control rats (Control, green dotted line with triangle) and rats administered with CFA (day 0) into the right (ipsilateral, CFA ipsi, red line with circle) hindpaw. The left hindpaw (contralateral, CFA contra, blue dotted line with square) was unhandled. Note that TWL of control animals and the contralateral hind paw of treated animals were parallel throughout the experimental design. CFA injection resulted in marked drop to 2.54 ± 0.22 s in TWL on post-injection day 3 [***p = (***p < 0.001)] at the ipsilateral hindpaws. The difference between data of the TWL obtained from control and CFA treated rats was also highly significant during experimental day 1–3 (***p < 0.001). Between-groups statistical differences were evaluated by using Repeated measures 2-way ANOVA followed by *Sidak’s* multiple comparison test. Data are shown as mean + SD.

The mechanical withdrawal threshold (MWT) values observed in control animals were comparable during the experimental duration, no significant differences were found between left and right hindpaws. The mean MWT (collected from the repeated measurements of left-and right hindpaw) was 49.47 ± 1.04 g in control animals. The contralateral (left, non-injected) hindpaw of the CFA treated animals also presented a similar mean MWT value (48.32 ± 1.96 g). However on the ipsilateral (right, CFA injected) hindpaw, provoked inflammation resulted in a significant decrease in MWT figures during the first three experimental days. The highest decline in MWT was detected on day 3 when it dropped to 20.6 ± 1.85 g (***p < 0.001). The difference between data of the MWT of control and CFA treated rats was strikingly significant during the first 3 days (***p < 0.001) (Figure 1A).

### 3.2. Animals showed significant thermal nociceptive sensitivity during peripheral inflammatory pain

The values of CFA evoked thermal allodynia were quite parallel to that of mechanical allodynia. Control-and contralateral (left, non-injected) hindpaws of CFA injected animals did not produce marked differences in TWL figures. The mean TWL values varied in the range of 5.57 ± 0.65–6.61 ± 0.63 s in control animals, whereas values between 5.92 ± 0.21–6.36 ± 0.77 s were recorded on the contralateral side of CFA treated rats. Regarding the ipsilateral (right, CFA injected) hindpaw, CFA injection also resulted in robust decrease. The highest drop in TWL was detected on day 3, when time values dropped to 2.54 ± 0.22 s (***p < 0.001). The discrepancy between data of the TWL obtained from control and CFA treated rats was also highly significant (***p < 0.001) (Figure 1B).

### 3.3. Spinal P2X4 gene-and protein expression were highly enhanced upon CFA injection

**Figure 2:**
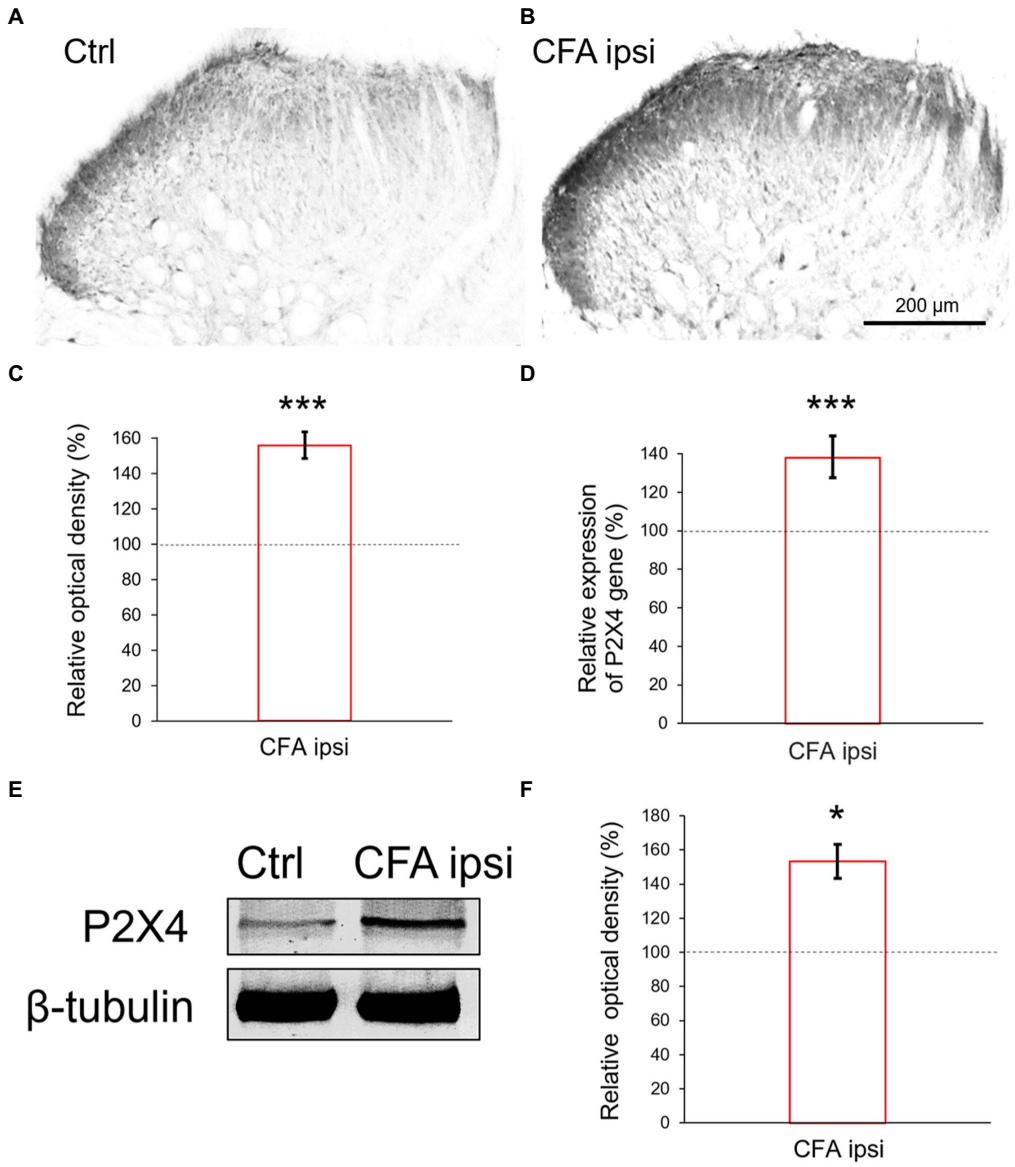
**(A,B)** Photomicrographs showing immunoperoxidase labelings for P2X4 within the spinal dorsal horn. In comparison with control animals **(A)** stronger P2X4 immunoreactivity was detected in Rexed laminae stronger P2X4 immunoreactivity was detected in Rexed laminae I and II of spinal cord sections taken from CFA treated animals **(B)**. Note that the intensity of the reaction was more prominent at the lateral aspect of the dorsal horn compared with medial areas. Scale bar: 200 μm. **(C)** Bar showing relative optical density measured from P2X4 immunoreactivity within the spinal dorsal horn during peripheral inflammation. The values represent the relative ratio of P2X4 expression (%) upon CFA treatment compared with control (100%). Inflammation substantially increased the P2X4 immunoreactivity (***p = 0.000501, 56% increase). Between-groups statistical differences were evaluated by Student’s t-test. Data are shown as mean + SD. **(D)** Bar showing P2X4 mRNA expression within the spinal dorsal horn in peripheral inflammation. The values represent the relative ratio of P2X4 mRNA (normalized to Rpl4 mRNA) upon CFA injection compared with control (100%). Inflammation substantially increased the amount of P2X4 transcripts (***p < 0.0001, 37.92% increase). Between-groups statistical differences were evaluated by Student’s t-test. Data are shown as mean + SD. **(E,F)** Representative blotting image and protein densitometry showing significant (*p = 0.013, 53.18% increase) enhancement of P2X4 protein in spinal dorsal horn tissue lysates (L4–L5) of animals 3 days following CFA injection (CFA ipsi) compared to control rats (Ctrl). β-tubulin was used as loading control. Between-groups statistical differences were evaluated by Student’s t-test. Data are shown as mean ± SD.

Our results showed an abundant immunoreactivity for P2X4 receptor in Rexed lamina I and II, whereas the deeper laminae were sparsely stained. Compared with control (Figure 2A) more robust P2X4 immunoreactivity (56 ± 7.42% increase, ***p = 0.000501) was obtained by densitometric analysis of spinal cord sections taken from CFA injected animals (Figures 2B,C).Subsequently, we aimed to elucidate whether the inflammation influenced the P2X4 expression at a transcriptional level. RT-qPCR experiments were designed from spinal dorsal horn tissue extracts of the L4-L5 segments. We found considerable enhancement of P2X4 mRNA transcripts (***p < 0.001, 37.92 ± 10.42% increase compared to control sample) at the summit of mechanical and thermal nociceptive sensitivity in inflammation (Figure 2D). We hypothesized that the tendency of P2X4 gene expression would be also explicit at a protein level, therefore protein blotting was conducted from spinal dorsal horn extracts of L4–L5 segments 3 days following CFA injection (Figure 2E; Supplementary Figure S2A). Densitometric analysis showed significant increase of the receptor protein in samples of CFA treated animals compared to control (53.18 ± 12.11% increase *p = 0.013) (Figure 2F).

### 3.4. P2X4 Receptor is upregulated on primary afferent fibers of spinal dorsal horn and lumbar DRG ganglia cells during peripheral inflammation

**Figure 3:**
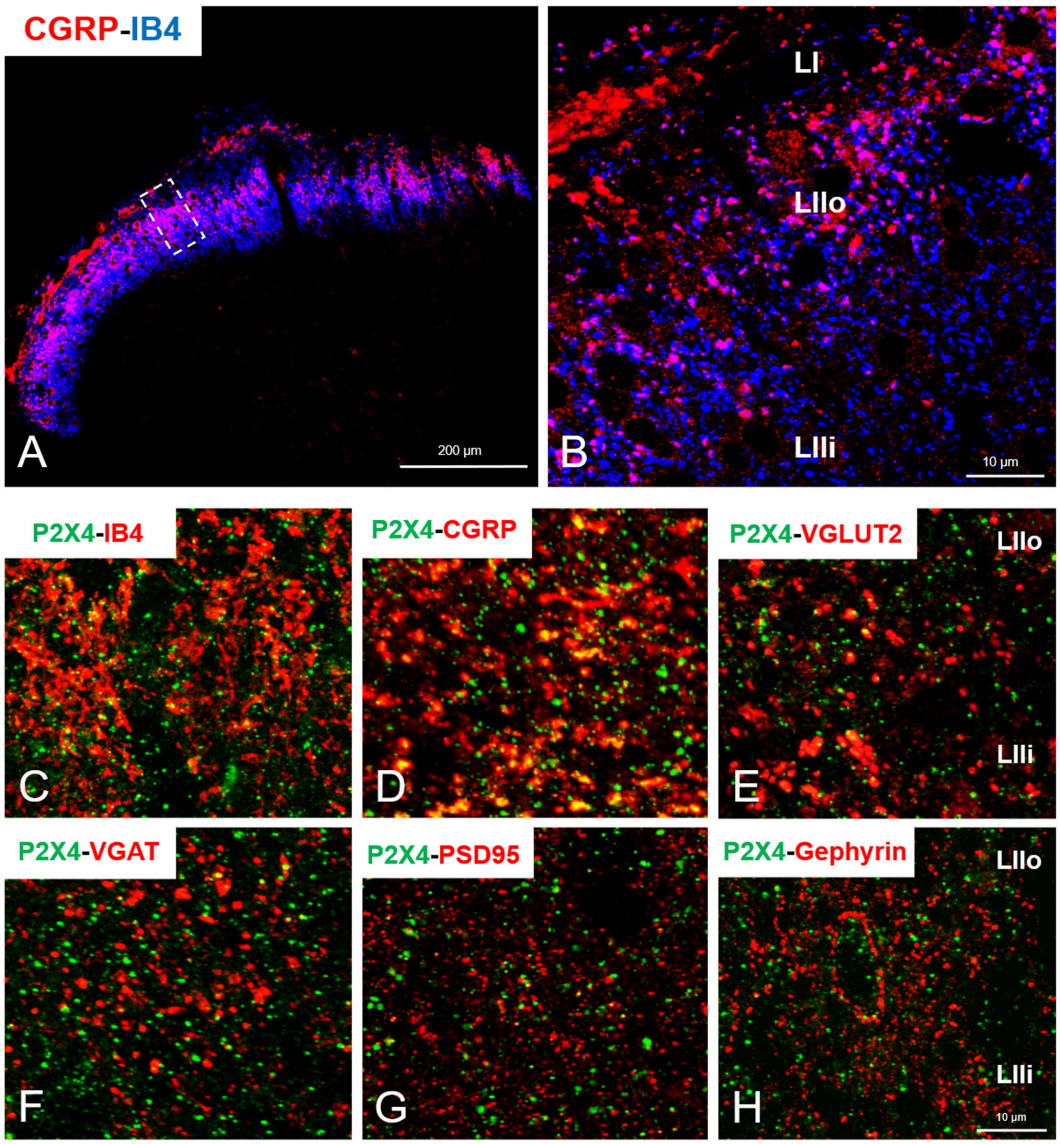
**(A)** Representative immunofluorescent double-labeled 1-μm thick confocal laser image showing CGRP (red color) and IB4 (blue color) immunoreactivity in the superficial spinal dorsal horn. **(B)** The area indicated by white rectangle with dashed line on panel A was further magnified. LI, LIIo and LIIi captions indicate the respective superficial laminae of Rexed within the spinal gray matter. Scale bars: 200 and 10 μm. **(C–H)** Representative immunofluorescent double-labeled 1-μm thick confocal laser images showing the colocalization of non-peptidergic and peptidergic primary afferents (IB4 and CGRP, red, **C,D**), excitatory and inhibitory interneurons- (VGLUT2 and VGAT, red, **E,F**) and excitatory and inhibitory postsynaptic markers (PSD95 and Gephyrin, red, **G,H**) with P2X4 receptor (green, **C–H**) within the superficial spinal dorsal horn of control animals. Scale bar: 10 μm.

**Figure 4:**
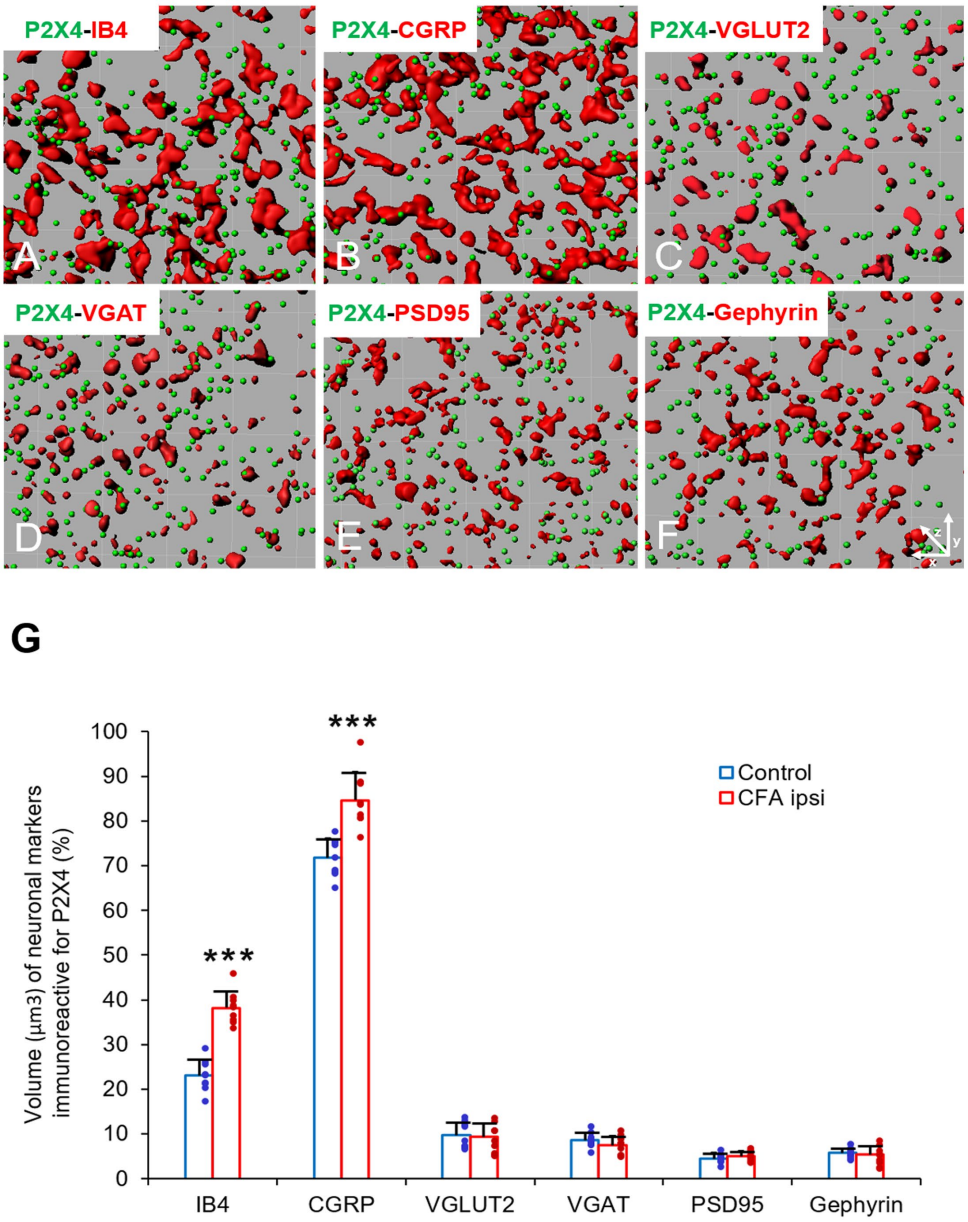
**(A–F)** Representative illustrations of double-labeled 1-μm thick confocal laser images rendered by IMARIS software. The illustrations represent IMARIS transformations (with x-z projections) of confocal images including presynaptic markers of non-peptidergic and peptidergic afferents (IB4 and CGRP, red, **A,B**), excitatory and inhibitory interneurons- (VGLUT2 and VGAT, red, **C,D**) and excitatory and inhibitory postsynaptic markers (PSD95 and Gephyrin, red, **E,F**) with P2X4 receptor (green, **A–F**) within the superficial spinal dorsal horn of control animals. **(G)** Bart chart showing the volume (μm^3^) of neuronal markers, which are immunoreactive for P2X4. Blue columns indicate data obtained from control animals (Control) whereas red columns indicate values calculated from CFA-treated animals (CFA ipsi). Individual datapoints of each column are also shown with colors (control, green dots, CFA ipsi-purple dots). Asterisks indicate that CFA-evoked inflammation significantly increased the volume of non-peptidergic primary afferent IB4 (***p = 0.0011, 15.16% increase) and peptidergic primary afferent CGRP (***p < 0.0001, 12.87% increase) immunoreactive puncta colocalized with P2X4 receptor. Data are shown as mean ± SD. Between-groups statistical differences were evaluated by Student’s *t*-test.

**Figure 5:**
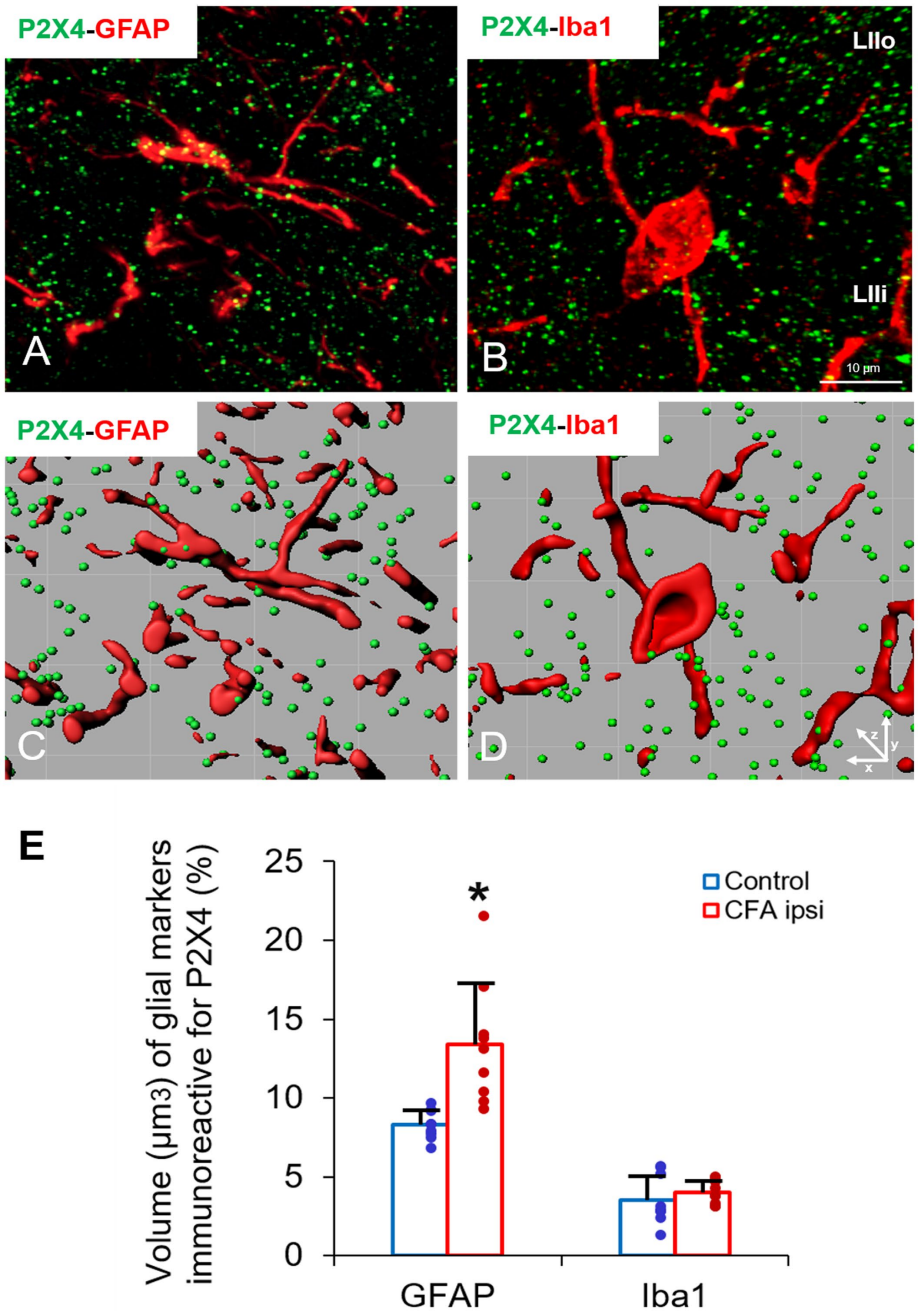
**(A–D)** Representative immunofluorescent double-labeled 1-μm thick confocal laser images supplemented by IMARIS analysis (with x-z projections) illustrating the colocalisation between immunoreactivity for markers that are specific to astrocytes (GFAP, red, **A,C**) or microglial cells (Iba1, red, **B,D**) and P2X4 receptor (green, **A–D**) in superficial spinal dorsal horn of control animals. Scale bar: 10 μm. **(E)** Bart chart showing the volume (μm^3^) of glial markers, which are immunoreactive for P2X4. Blue columns indicate data obtained from control animals (Control) whereas red columns indicate values calculated from CFA-treated animals (CFA ipsi). Individual datapoints of each column are also shown with colours (control, green dots, CFA ipsi-purple dots). Asterisks indicate that CFA-evoked inflammation moderately increased the volume of GFAP positive astrocytes (*p = 0.0103, 5.1% increase) colocalised with P2X4 receptor. Iba1 positive microglial cells showed significant P2X4 receptor expression neither in control nor upon CFA treatment. Data are shown as mean ± SD. Between-groups statistical differences were evaluated by Student’s *t*-test.

**Figure 6:**
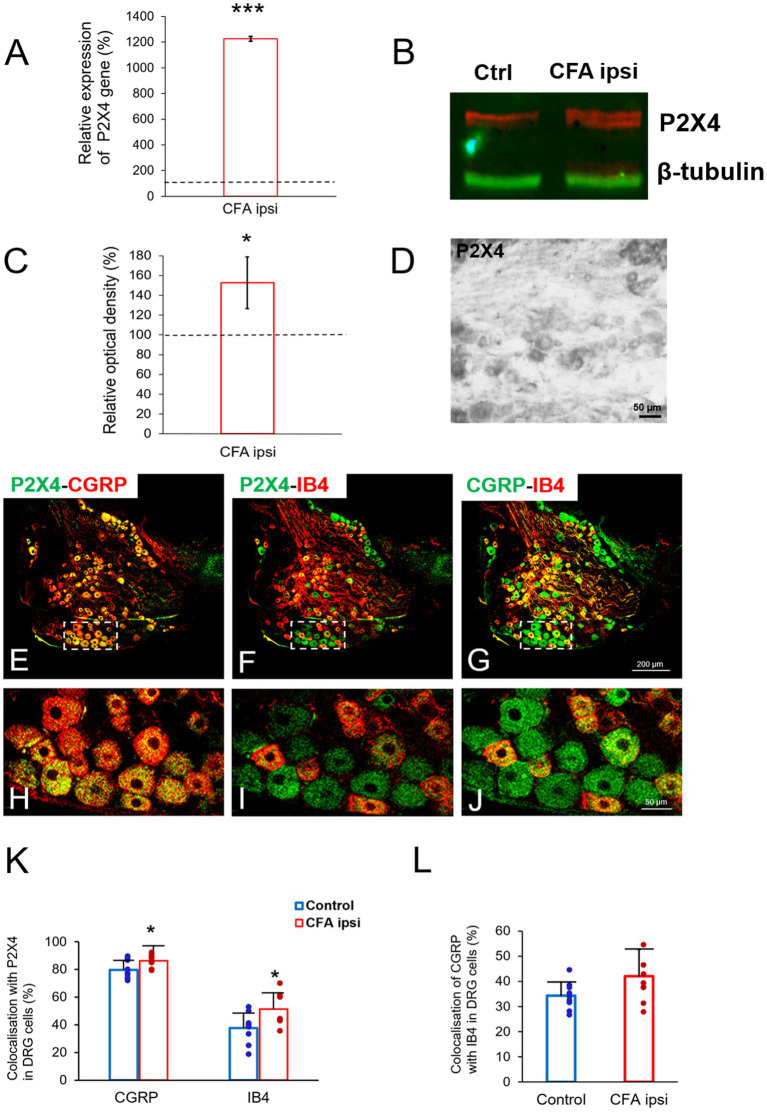
**(A)** Bar chart showing P2X4 mRNA expression (%) within lumbar DRG tissue (L4–L5) in peripheral inflammatory pain. Values represent the relative ratio of P2X4 mRNA (normalized to Rpl4 mRNA value) upon CFA treatment compared with control. CFA injection robustly increased the amount of P2X4 mRNA transcripts (***p = 0.0005, 1,224% increase). Between-groups statistical differences were evaluated by Student’s t-test. Data are shown as mean + SD. **(B,C)** Representative western blot image and protein densitometry showing significant enhancement of P2X4 protein (*p = 0.05, 52.7% increase) in lumbar DRG tissue (L4-L5) lysates of animals 3 days following CFA injection (CFA ipsi) compared to control rats (Ctrl). β-tubulin was used as loading control. Between-groups statistical differences were evaluated by using Student’s *t*-test. Data are shown as mean + SD. **(D)** Photomicrograph showing immunoperoxidase labeling for P2X4 within lumbar DRG section (L4) of control animal. Scale bar: 50 μm. **(E–J)** Representative immunofluorescent double-labeled 1-μm thick confocal laser images of lumbar DRG tissue (L4–L5) showing colocalisation of either peptidergic or non-peptidergic nociceptive neuronal profiles (CGRP, red; **E,H**, IB4, red; **F,I**, respectively) with P2X4 receptor (green, **E, F, H, I**) as well as colocalisation of CGRP with IB4 marker **(G–J)** in control animals. White rectangles with dashed line of panels **E–G** were magnified on panel **H–J**. Scale bars: 200 **(G)** and 50 μm (J). **(K–L)** Bar chart showing colocalization values (%) of peptidergic and non-peptidergic lumbar DRG (L4–L5) profiles (CGRP and IB4) with P2X4 receptor as well as the colocalization of CGRP with IB4 marker. Blue columns indicate data obtained from control animals (Control) whereas red columns indicate values from CFA-treated animals (CFA ipsi). Individual datapoints of each column are also shown with colors (control, green dots, CFA ipsi-purple dots). Asterisks indicate that CFA-evoked inflammation significantly increased colocalization of CGRP and IB4 positive ganglion cells (*p = 0.0263 6.48% increase, and *p = 0.0245 13.77% increase respectively) with P2X4 receptor, however CFA injection did not influence overlap between the markers. Between-groups statistical differences were evaluated by Student’s *t*-test. Data are shown as mean ± SD.

In our study we aimed to decipher the fine spinal distribution of P2X4 in peripheral inflammatory pain. Thus, we carried out double immunostainings in which we determined the localization of P2X4 immunoreactive spots on neurons and glial cells of the superficial spinal dorsal horn by IMARIS analysis. Superficial Rexed laminae I and II of spinal dorsal horn for colocalization analysis were distinguished by performing double immunostaining against CGRP and IB4, where CGRP labelled Rexed lamina I and IIo, and IB4 indicated Rexed lamina IIi (Figures 3A,B). To determine whether the receptor was present on central axonterminals including non-peptidergic and peptidergic nociceptive primary afferents as well as axonterminals of glutamatergic and GABAergic interneurons, we analyzed the colocalization of selected markers (IB4, CGRP, VGLUT2 and VGAT) with P2X4. Based on our findings 23.08 ± 3.54% of the IB4 positive non-peptidergic fibers certain to be positive for P2X4 receptor in control animals, which significantly increased (***p = 0.0011) to 38.24 ± 3.67% (Figures 3C, 4A,G) in CFA administered rats. 71.79 ± 4.25% of CGRP positive peptidergic primary afferent fibers showed colocalization with P2X4 in control animals which also significantly (***p < 0.001) raised to 84.66 ± 6.24% in peripheral inflammatory pain (Figures 3D, 4B,G). VGLUT2 positive excitatory-and VGAT positive inhibitory axonterminals of spinal interneurons showed no significant change in P2X4 expression upon CFA treatment. In control animals 9.67 ± 2.92% of the VGLUT2 positive fibers were colocalized with P2X4, whereas in inflammatory pain it was calculated as 9.34 ± 3.1% (Figures 3E, 4C,G). In case of VGAT positive fibers we observed similar results, between-groups difference was statistically irrelevant. 8.61 ± 1.67% of the axonterminals overlapped with P2X4 receptor in control rats, whereas upon CFA injection 7.47 ± 2.1% was obtained (Figures 3F, 4D,G). In contrast to results obtained from spinal axonterminals, on postsynaptic dendrites lower receptor number was detected. 4.54 ± 1.15% of PSD95 positive excitatory postsynaptic dendrites showed positivity for P2X4 in control conditions, and CFA treatment did not influence the receptor expression (4.99 ± 1.09%) (Figures 3G, 4E,G). Inhibitory postsynaptic density marker gephyrin colocalized with P2X4 in the same degree. 5.84 ± 0.95% of gephyrin positive dendrites were also immunoreactive for P2X4 in control animals, which was calculated as 3.68 ± 0.16% in CFA model (3.68 ± 0.16%) (Figures 3H, 4F, G). Astrocytes abundantly expressed P2X4 during inflammatory pain. 8.28 ± 0.93% of GFAP-labelled astrocyte membrane compartments were found to express the receptor in control conditions, which were increased to 13.38 ± 3.9% upon CFA administration (*p = 0.0103) (Figures 5A,C,E). Intriguingly, microglial cells in our model did not contribute to P2X4 receptor upregulation in inflammation. 3.5 ± 1.56% of Iba1 positive microglial cells showed positivity for P2X4 in control animals, which was unchanged upon CFA injection (3.99 ± 0.75%) (Figures 5B,D,E). In lumbar DRG tissue (L4-L5) the amount of P2X4 gene transcripts was exceptionally increased (***p = 0.0005) upon CFA treatment (1,224.40 ± 19.37%, more than 12 times fold change increase compared to control) (Figure 6A). This finding was moderately supported by protein blotting, which verified notable increase (52.77 ± 44.77% increase compared to control, *p = 0.05) (Figures 6B,C; Supplementary Figure S2B). Single immunoperoxidase labeling was also performed to visualize the receptor expression within L4-L5 DRG cells (Figure 6D). Evaluation of P2X4 colocalization with nociceptive markers (CGRP, IB4) of small diameter neurons proposed that in control animals 79.76 ± 6.76% of the peptidergic CGRP positive neuronal profiles coexpressed P2X4 receptor, which was enhanced to 86.24 ± 4.42% upon CFA treatment based on IMARIS calculation (*p = 0.0263) (Figures 6E,H,K). In case of non-peptidergic IB4 positive cells 37.65 ± 10.87% was colocalized with P2X4 receptor, this ratio was also substantially elevated in inflammatory pain (51.42 ± 11.71%, p* = 0.0245) (Figures 6F,I,K). We also analyzed the putative overlapping between CGRP and IB4 positive neuronal profiles. 34.27 ± 5.45% of CGRP positive cells showed positivity for IB4, which was insignificantly changed to 41.99 ± 10.50% in peripheral inflammation (Figures 6G,J,L).

### 3.5. Ultrastructural analysis clarifies the localization of P2X4 On axonal profiles and neuronal soma

**Figure 7:**
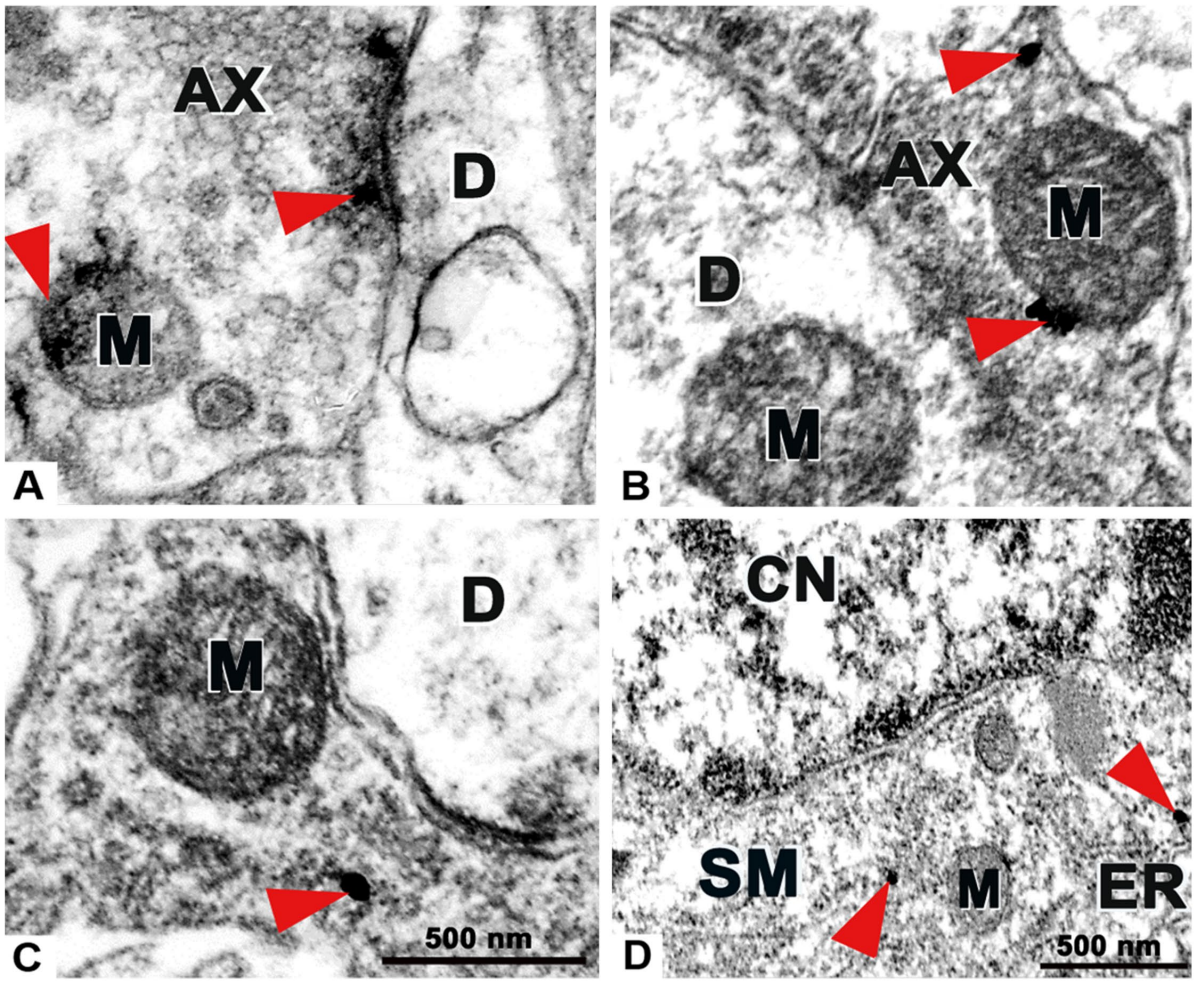
Transmission electron microscopy images demonstrating the ultrastructure of the superficial spinal dorsal horn with preembedding immunolabeling for P2X4 receptor. **(A)** Example of an immunoperoxidase reaction for P2X4 on an axon profile (AX). Red arrows show clusters of immunoprecipitates accumulated at the proximity of the presynaptic membrane and mitochondrium (M). No precipitate was detected on dendrite (D). **(B,C)** Examples of nanogold immunolabelings for P2X4 on axon profiles (AX). Red arrows show deposits of gold nanoparticles intensified with silver on synaptic axonterminal near mitochondrium (M), dendrites (D) were not stained. **(D)** Nanogold immunolabeling for P2X4 on soma (SM). Red arrows show nanoparticles within the cytoplasm adjacent to endoplasmatic reticule (ER) and mitochondria (M) at the proximity of cell nucleus (CN). Scale bars: 500 nm.

We intended to provide novel experimental evidence regarding the ultrastructural distribution of P2X4 receptor by nanogold labeling within superficial spinal dorsal horn. Both pre-and postsynaptic regulation were earlier suggested for P2X4 based on ultrastructural results of Lê et al. (1998), and in fact PSD95 and gephyrin markers colocalized with the receptor in some extent, still our results rather emphasize the presynaptic role of spinal P2X4 receptor. Immunoperoxidase reaction verified clusters of immunoprecipitates at the synaptic vesicles as well as near the presynaptic contacts and mitochondrium (Figure 7A). These observations were confirmed by nanogold labelings, deposits of silver intensified gold nanoparticles were accumulated at the proximity of presynaptic membrane, synaptic vesicles and mitochondria (Figures 7B,C). In addition, in agreement with previous findings of Lê et al. (1998) we also detected gold nanoparticles on neuronal soma adjacent to endoplasmatic reticule and mitochondria (Figure 7D).

## 4. Discussion

In this study we elucidated the expression and cellular distribution of P2X4 receptor within the spinal dorsal horn and lumbar DRG cells of L4-L5 segments in control and CFA treated male rats. Validity of CFA model concept as well as mechanical and thermal behavioral tests were previously confirmed by Molander and Grant (1985), Ma and Woolf (1996), Raghavendra et al. (2004). Briefly, according to our results significant increase in the level of P2X4 mRNA was detected within the spinal dorsal horn 3 days following the CFA administration. Immunoperoxidase technique also showed robust enhancement of P2X4 immunoreactivity within Rexed laminae I and II of the spinal dorsal horn, which was further supported by Western blot. With IMARIS, a major fraction of P2X4 receptor has been clarified on primary afferent axonterminals and astrocytes, despite its expression on presynaptic axons and neuronal somata, it was absent on postsynaptic dendrites when validated at ultrastructural level. The L4-5 DRG analysis also showed increased expression of P2X4 in peptidergic CGRP-positive and non-peptidergic IB4 nociceptive subsets of ganglia cells after CFA injection. Of note, CGRP and IB4 positive subsets of DRG cells have considerable overlap, which was earlier described in rats by multiple investigations (Petruska et al., 2002; Price et al., 2005; Price and Flores, 2007). It has been well documented that nerve injury upregulates spinal microglial P2X4 expression (Ulmann et al., 2008; Tsuda et al., 2013; Stokes et al., 2017; Inoue, 2019). In inflammatory and neuropathic pain models (Todd, 2010; Aby et al., 2018; Peirs et al., 2020) increased excitability was reported within the superficial spinal dorsal horn. Besides, it has been recently reported that P2X4 receptor contributes to several neuropathological states as it modulates synaptic transmission altering hippocampal memory *via* downregulation of GABA A receptor (Roshani et al., 2022), but its increased surface expression was also detected in mice suffering from amyotrophic lateral sclerosis (Bertin et al., 2022). In addition, P2X4 deficient mice showed impaired inflammasome function upon spinal cord injury (de Rivero Vaccari et al., 2012). P2X4 inhibition was also found to reduce microglial directed inflammation and apoptosis *via* Nod-like receptor protein 3 (NLRP3) inflammasome in rat brain trauma model (He et al., 2022). Microglial P2X4 activation elicits brain-derived neurotrophic factor (BDNF) release, which downregulates neuronal K^+^-Cl^−^cotransporter 2 (KCC2). As a result, polarity switch of inhibitory GABA-and glycinergic iongradient leads to excitatory activation of the spinal dorsal horn network (Coull et al., 2005; Montilla et al., 2020). Thus, P2X4-BDNF signaling is relatively well understood in neuropathic pain. Genetic depletion of microglial derived BDNF prevents the development of neuropathic allodynia. Interestingly, major gender differences were observed in P2X4 signaling of chronic neuropathic pain, hence microglial cells were found to be dispensable for the establishment of allodynia in female, but not in male animals. In the former, most probably adaptive immune cells contribute to spinal pain signaling (Sorge et al., 2015; Malcangio, 2017; Mapplebeck et al., 2018; Khir et al., 2021). However, in peripherally induced inflammation P2X4 signaling may differ. Lalisse et al. (2018) found earlier in CFA model that BDNF evoked KCC2 downregulation did not depend on microglial cells. Supposedly, P2X4 receptor upregulation elicits BDNF production of lumbar DRG cells in order to be transported *via* primary central axonterminals for secretion within spinal dorsal horn in a neuronal guided manner (Cho et al., 1997; Lever et al., 2001; Lalisse et al., 2018). Moreover, selective genetic deletion of BDNF in Nav1.8 positive sensory neurons also reduced inflammation without influencing neuropathic sensitivity (Zhao et al., 2006). Till date scanty and contradictory information are available about the P2X4 expression in lumbar DRG tissue upon inflammation, hence others also documented that CFA did not alter P2X4 expression in lumbar DRGs, but did in spinal cord (Xu et al., 2018). Earlier studies reported substantial expression of P2X4 receptor in several types of ganglia cells (Bo et al., 2003; Zhang et al., 2020) and macrophages in inflammation (Ulmann et al., 2010). Our results may also shade these postulations. In our samples P2X4 mRNA expression was 12 times larger than that of the control after CFA injection, which was also consistent with data retrieved from studies related to neuropathia (Deng et al., 2018; Kohno and Tsuda, 2021), and western blot experiments, even if the enhancement of P2X4 protein expression proved to be rather moderate in comparison with the mRNA profile. The differences between mRNA and protein amount may be explained by posttranscriptional mechanisms that control protein levels regardless of mRNA amount (McCarthy, 1998; Csárdi et al., 2015; Brion et al., 2020; Buccitelli and Selbach, 2020). In diabetic neuropathic model, immunofluorescent labelings showed predominant P2X4 expression by satellite cells (Teixeira et al., 2019), but in our model satellite cells were not found to be stained with P2X4, instead we rather highlighted that CGRP and IB4 positive subsets of lumbar ganglia cells were substantially colocalized with P2X4 receptor, but change in overlap between CGRP and IB4 markers was found to be insignificant in peripheral inflammation. Interestingly, CFA treatment did not alter the amount of P2X4 receptor within lumbar DRG cells implying that peripheral inflammation may influence exclusively the spinal P2X4 expression (Xu et al., 2018). The controversial data between CFA models may be explained either with different species (mice vs. rats), or the time-course of the experimental design selected by the different research groups. Moreover, the degree of swelling and hypersensitivity may be correlated with the way of CFA preparations and injections administered (McCarson and Fehrenbacher, 2021). P2X4 shows a widespread distribution, throughout the central and peripheral nervous system. Beyond microglial cells, the receptor has been already detected in neurons including but not limited to olfactory bulb, hypothalamus, cerebellum (Collo et al., 1996) substantia nigra (Amadio et al., 2007), retinal ganglion cells (Wheeler-Schilling et al., 2001), and spinal dorsal horn (Bardoni et al., 1997). However, still little is known about the precise neuronal localization of spinal P2X4. Overexpression of P2X4 receptor may directly facilitate the responsiveness of the neuronal network within the spinal dorsal horn resulting in inflammation. *In vivo* extracellular recordings demonstrated CFA induced hyperexcitability of wide dynamic range (WDR) neurons within murine spinal dorsal horn *via* decrease of the C-fiber response threshold. C-fibers are connected with WDR neurons of the superficial laminae of spinal dorsal horn, where increase of wind-up amplitude measured in inflammatory pain, is robustly suppressed in P2X4 −/− mice (Aby et al., 2018). P2X4 activation may control gene expression *via* Ca^2+^ signaling, and promotes neurotransmitter release to enhance sensory transmission that eventually leads to central sensitization associated with inflammatory pain. Furthermore, in P2X4R deficient mice following peripheral inflammation BDNF signaling as well as extracellular signal-regulated kinase (ERK) regulated phosphorylation of N-metyl-D-aspartate (NMDA) receptor subunit GluN1 and KCC2 downregulation were all attenuated, proposing a significant role for P2X4 receptor in chronic inflammation (Lalisse et al., 2018; Khir et al., 2021). We also detected robust increase of P2X4 mRNA and protein level in the spinal dorsal horn at post-injection day 3. Peripheral inflammation resulted in highly significant P2X4 enrichment on non-peptidergic IB4 and peptidergic CGRP positive primary afferent fibers, concurrently VGLUT2 positive profiles were only weakly colocalized with the receptor. We contemplated that there is a great deal of interest in this matter, hence as reported by Li et al. (2003) significant numbers of VGLUT2 labeled axonterminals found in the spinal dorsal horn were of primary afferent origin following dorsal rhizotomy. Thus, quantitative data regarding P2X4 expression of CGRP and IB4 labeled primary afferents should also include data related to VGLUT2 positive terminals. Nevertheless, the background of this question is much more complex than initially thought, hence based on earlier results of Todd et al. (2003) peptidergic axonterminals labeled with either CGRP and substance P or CGRP and somatostatin antibodies were either weakly immunoreactive or not immunoreactive for VGLUT2. Furthermore, those nonpeptidergic fibers that were labelled with biotinylated IB4 also showed either weak positivity for VGLUT2 or were not immunostained. In contrast, high VGLUT2 expression was detected in majority of CGRP and IB4 positive somata of DRG cells (Brumovsky, 2013). These findings were corroborated by other authors such as Alvarez et al. (2004), who also revealed low level of VGLUT2 expression in central terminals of nociceptors by performing dorsal rhizotomy. They concluded that VGLUT2 immunofluorescence was not substantially reduced upon rhizotomy, therefore the majority of VGLUT2 positive immunoreactivity should originate from intrinsic source of the spinal dorsal horn. Moreover, authors proposed that probably the signal of primary afferents was abundantly outnumbered by strong VGLUT2 immunoreactivity of the intrinsic spinal interneuron terminals, hence the resolution of their methods was not enough to perform quantitative analysis. We also propose as a possible explanation that robust VGLUT2 expression of spinal dorsal horn neurons might mask changes occurring in VGLUT2 positive primary afferent terminals. Differences between the studies may be due to the different VGLUT2 antibodies used, hence Li et al. used their specially manufactured affinity-purified antibodies for VGLUT transporters. Our workgroup applied one of the same antibodies for VGLUT2 (guinea pig anti-vesicular glutamate transporter 2 (VGLUT2, 1:2000, Chemicon/Millipore, Temecula California, USA, catalog no.: AB2251), which was used by Todd and Alvarez. Thus, based on their earlier observations, in our experimental design CGRP or IB4 positive primary afferent fibers were regarded as VGLUT2 negative. The distribution of the P2X4 receptor was also verified on glial cells and neuronal somata. Unfortunately, still little is revealed regarding the specific role of P2X4 in glial cells other than microglia (Montilla et al., 2020). Regarding our findings from CFA model, we suppose that rather astrocytes than microglial cells contribute to P2X4 expression, whereas we do not have experimental data on oligodendrocytes. Accumulating data indicate the putative interaction between P2X4 and P2X7 receptors. Supposedly, P2X4 promotes P2X7 receptor directed NLRP inflammasome activation, resulting in production of IL-1ß and IL-18 (Kanellopoulos et al., 2021). Interestingly, our earlier results (Ducza et al., 2021) showed increased amount of NLRP2 protein in astrocytes of the spinal dorsal horn upon CFA injection, which puts our latest finding about the significant overexpression of P2X4 receptor on astrocytes into a new perspective. Nevertheless, there is still no experimental evidence regarding the correlation between P2X4 receptor and NLRP2 inflammasome. P2X4 receptor expression within neuronal soma was not surprising, hence earlier data suggested the existence of a permanent and dynamic turnover between cell membrane and intracellular organelles (Royle et al., 2005; Qureshi et al., 2007; Cao et al., 2015) thus there may be a constant P2X4 trafficking between neuronal plasmamembrane and intracellular organelles as well, which regulates receptor density as well as sensitisation-desensitization cycles (Robinson and Murrell-Lagnado, 2013; Xu et al., 2014). Interestingly, the receptor fraction is negligible on excitatory and inhibitory postsynaptic dendrites. This notion implies that even if purinergic signaling is identified both at pre-and postsynaptic sites by Lê et al. (1998), here we rather emphasize the former, hence at a spinal level rather the presynaptically located P2X receptors had drawn attention earlier by inducing glutamate release from sensory neurons (Gu and MacDermott, 1997; Montilla et al., 2020) and regulating synaptic co-transmission of molecules in cultured rat dorsal horn neurons (Hugel and Schlichter, 2000; Montilla et al., 2020).

## 5. Conclusion

We concluded that, lumbar DRG-and spinal overexpression of P2X4 receptor were tangible at a gene-and protein level in peripheral inflammation, moreover, we propose here that IB4 and CGRP positive DRG neurons and primary afferent terminals may considerably contribute to P2X4 dependent spinal pain signalization.

## Data availability statement

The original contributions presented in the study are included in the article/supplementary material, further inquiries can be directed to the corresponding author/s.

## Ethics statement

The animal study was reviewed and approved by Animal Welfare Committee of the University of Debrecen.

## Author contributions

KHo and LD: conceptualization. LD prepared the manuscript, performed immunohistochemical stainings and quantitative IMARIS analysis. LD and KHe carried out CFA injections and behavioral tests. KHo, BG, KM, and PS edited and reviewed the manuscript. AG and GK photographed the confocal-and electronmicroscopical sections. RT conducted and analyzed qPCR experiments. KHo and EB carried out western blotting and data analysis. All authors contributed to the article and approved the submitted version.

## Funding

This project was supported by the Hungarian National Brain Research Program (nos. KTIA_NAP_13-2-2014-0005 and 2017–1.2.1-NKP-2017-00002).

## Conflict of interest

The authors declare that the research was conducted in the absence of any commercial or financial relationships that could be construed as a potential conflict of interest.

## Publisher’s note

All claims expressed in this article are solely those of the authors and do not necessarily represent those of their affiliated organizations, or those of the publisher, the editors and the reviewers. Any product that may be evaluated in this article, or claim that may be made by its manufacturer, is not guaranteed or endorsed by the publisher.

## Footnotes

^1^ https://www.iasp-pain.org/resources/terminology/

^2^ https://us.vwr.com/store/product/21762277/tween-tris-buffered-saline-ttbs-10x-concentrate-ph-7-5-rockland

## Abbreviations

BDNF: brain-derived neurotrophic factor
CFA: complete Freund adjuvant
CGRP: calcitonin gene-related peptide
DRG: dorsal root ganglion
GABA: γ-aminobutyric acid
GFAP: glial fibrillary acidic protein
Iba1: ionized calcium binding adaptor molecule 1
IB4: isolectin B4
MWT: mechanical withdrawal threshold
NGS: normal goat serum
NLRP: Nod-like receptor protein
NMDA: N-metyl-D-aspartate receptor
P2X4: P2X purinoreceptor 4
PBS: phosphate-buffered saline
PSD95: postsynaptic density protein 95
RPL4: ribosomal protein L4
SD: standard deviation
SEM: standard error of mean
TPBS: Tris-phosphate-buffered saline
TWL: thermal withdrawal threshold
VGLUT2: vesicular glutamate transporter 2
VGAT: vesicular gamma-amino butyric acid transporter
