# Supplementary figures and images for: Neuronal P2X4 receptor may contribute to peripheral inflammatory pain in rat spinal dorsal horn
Source: Front Mol Neurosci. 2023 Mar 9;16:1115685. doi: 10.3389/fnmol.2023.1115685 (PMC10033954; doi:10.3389/fnmol.2023.1115685)

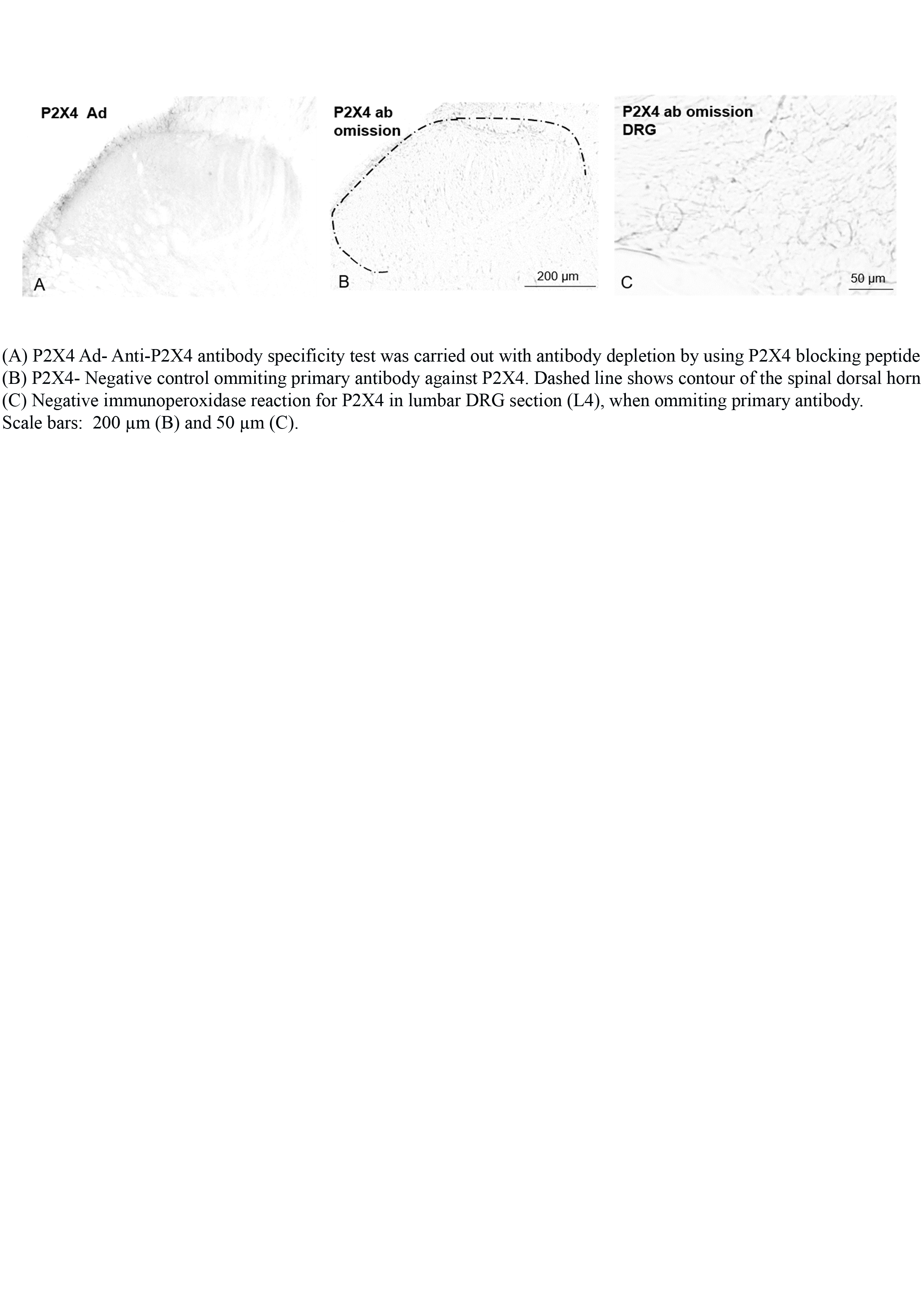

Supplement: Supplementary file 1 [file Image_1.TIF]

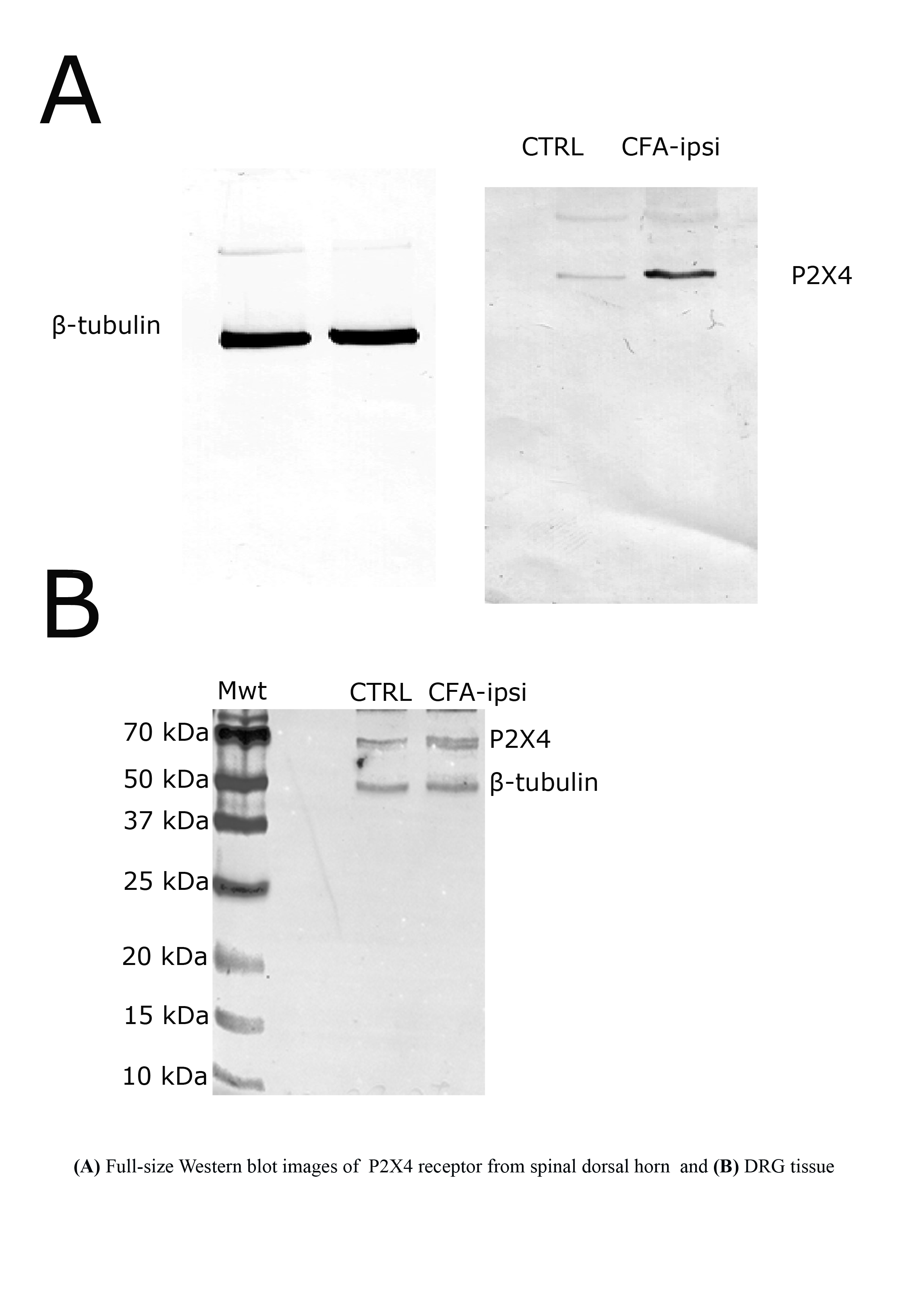

Supplement: Supplementary file 2 [file Image_2.TIF]
