# Supplementary material for: Neuronal P2X4 receptor may contribute to peripheral inflammatory pain in rat spinal dorsal horn
Source: Front Mol Neurosci. 2023 Mar 9;16:1115685. doi: 10.3389/fnmol.2023.1115685 (PMC10033954; doi:10.3389/fnmol.2023.1115685)
Supplement: Supplementary file 3 [file Table_1.docx]

**Table S1.** Nucleotide sequences, amplification sites, GenBank accession numbers and amplicon sizes for each rattus norvegicus qPCR primer pair are shown. The temperature for annealing/extension was 60 °C in all cases.

| ***Gene*** | ***Primer*** | ***Nucleotide sequence (5’→3’)*** | ***GenBank ID*** | ***Amplicon size (bp)*** |  |
| --- | --- | --- | --- | --- | --- |
| *Purinergic receptor* | | |  |  |  |
| **P2rx4** | sense  antisen  se | CCT TCC TGT TCG AGT ACG ACA  (129–149)  ACG AAC ACC CAC CCG ATG A  (246–228) | **NM_031594.2** | 118 |  |
| *Reference genes* | | |  |  |  |
| **Ywhaz** | sense  antisense | TCT GCA ACG ACG TAC TGT CTC  (339–359)  CCT CAG CCA AGT AGC GGT AG  (455–436) | **NM_013011.4** | 117 |  |
| **Ppia** | sense  antisense | GAC GCC GCT GTC TCT TTT CG  (8–27)  TTT GTC TGC AAA CAG CTC GAA G  (126–105) | **NM_017101.1** | 119 |  |
| **Rplp0** | sense  antisense | CCC ACT GGC TGA AAA GGT CAA G  (850–871)  CTG ACT TGG TGT GAG GGG CT  (1046–1027) | **NM_022402.2** | 197 |  |
| **Rpl4** | sense  antisense | CGC CAG GCT AGG AAT CAC AAA  (1075–1095)  TGG CAT CCA CAG GCT TCT TT  (1225–1206) | **NM_022510.1** | 151 |  |
