# Supplementary material for: Neuronal P2X4 receptor may contribute to peripheral inflammatory pain in rat spinal dorsal horn
Source: Front Mol Neurosci. 2023 Mar 9;16:1115685. doi: 10.3389/fnmol.2023.1115685 (PMC10033954; doi:10.3389/fnmol.2023.1115685)
Supplement: Supplementary file 4 [file Table_2.docx]

**TABLE S2.** List of primary and secondary antibodies

| **Primary antibody** | **Dilution** | **Distributor** | **Catalog no.** |
| --- | --- | --- | --- |
| Anti-P2X4 | 1:1000 | Alomone Labs | APR-002 |
| Anti-CGRP | 1:2000 | Peninsula Labs | T-5027 |
| IB4 | 1:2000 | Invitrogen/ Thermofisher | I21414 |
| Anti-VGLUT2 | 1:2000 | Merck Millipore | AB2251 |
| Anti-VGAT | 1:200 | Synaptic Systems | 131011 |
| Anti-PSD95 | 1:100 | Frontier | AB2327 |
| Anti-gephyrin | 1:100 | Synaptic Systems | 147021 |
| Anti-GFAP | 1:1000 | Merck Millipore | MAB3402 |
| Anti-Iba1 | 1:2000 | Synaptic Systems | 234-004 |
| **Secondary antibody** | **Dilution** | **Distributor** | **Catalog no.** |
| GAR conjugated with Alexa Fluor488 | 1:1000 | Thermofisher | A11034 |
| GAGp conjugated with Alexa Fluor555 | 1:1000 | Thermofisher | A21435 |
| Streptavidin conjugated with Alexa Fluor555 | 1:1000 | Thermofisher | S21381 |
| GAM conjugated with Alexa Fluor555 | 1:1000 | Thermofisher | A21422 |
